# Strengthening cancer care for the LGBTQIA+ population in Nepal: A narrative review to set priorities for equitable oncology services^☆^

**DOI:** 10.1016/j.dialog.2025.100229

**Published:** 2025-07-05

**Authors:** Sunil Shrestha, Nabin Pathak, Simit Sapkota, Sudip Thapa, Subhas Pandit, Jeebana Bhandari, Pankaj Barman, Pratik Khanal, Kamal Ranabhat, Vibhu Paudyal, Deependra Singh

**Affiliations:** a Department of Research and Academics, Kathmandu Cancer Center, Nala Road, Tathali, Bhaktapur, Nepal; b Drug Information Unit and Pharmacovigilance Cell, Department of Pharmacy, Hetauda Hospital, Madan Bhandari Academy of Health Sciences, Hetauda, Bagmati Province, Nepal; c Department of Pharmacy and Clinical Pharmacology, Madan Bhandari Academy of Health Sciences, Hetauda, Bagmati Province, Nepal; d Department of Clinical Oncology, Kathmandu Cancer Center, Nala Road, Tathali, Bhaktapur, Nepal; e Department Medical Oncology, B&B Hospital Pvt. Ltd, Gwarko, Lalitpur, Nepal.; f Chitwan Medical College, Department of Medical Oncology, Chitwan, Nepal; g Bergen Centre for Ethics and Priority Setting in Health (BCEPS), Department of Global Public Health and Primary Care, University of Bergen, Norway; h Ministry of Health and Population, Government of Nepal, Kathmandu, Nepal; i Institute of Medicine, Tribhuvan University, Kathmandu, Nepal; j Florence Nightingale Faculty of Nursing, Midwifery and Palliative Care, King's College London, London, UK; k Early Detection, Prevention, and Infections Branch, International Agency for Research on Cancer (IARC/WHO), Lyon, France

**Keywords:** LGBTQIA+, cancer care, Oncology, Stigma and barriers, Equitable healthcare, Nepal

## Abstract

Cancer epidemiology and care services in low- and middle-income countries, has traditionally overlooked the specific needs of the lesbian, gay, bisexual, transgender, queer, intersex, and asexual community, including people who identify with diverse gender identities and sexual orientations (LGBTQIA+). This narrative review examines the intersection of LGBTQIA+ individual's health and oncology cases in Nepal, highlighting disparities in cancer risk factors, delayed diagnosis, limited screening access and the compounding effects of social stigma and discrimination. Drawing from regional data and global insights, we identify systemic barriersincluding heteronormative healthcare environments, lack of provider training in LGBTQIA+-inclusive oncology, and policy gaps that hinder equitable cancer care access. We also outline targeted strategies to improve cancer outcomes for LGBTQIA+ individuals, including stakeholder engagement, culturally competent oncology training for healthcare providers and students, and community-led education and advocacy. This review underscores the urgent need to integrate LGBTQIA+-specific priorities into Nepal's national cancer strategies to advance equity in oncology care delivery.

## 1 Introduction

Cancer is a major public health issue, with substantial morbidity and mortality worldwide. In 2022, the World Health Organization (WHO) estimated 20 million new cancer cases and nearly 10 million cancer-related deaths globally, with the burden expected to rise significantly by 2050 [1,2]. This growing burden disproportionately impacts vulnerable populations, including those with limited access to healthcare services [3,4] along with timely diagnosis, treatment, and palliative care [5]. Among these populations, individuals who identify as lesbian, gay, bisexual, transgender, queer, intersex, asexual and other diverse sexual orientations and gender identities (LGBTQIA+) face distinct and often overlooked challenges in accessing equitable cancer care [3,4].

In low-middle-income countries (LMIC) such as Nepal, these challenges are compounded by societal norms, limited healthcare resources, limited access to healthcare services, and inequitable access to services. Despite progressive steps, such as the legal recognition of a “third gender” in 2007, same-sex marriage (December 21st, 2007) and the subsequent inclusion of this category in voting polls and the national census, LGBTQIA+ individuals continue to face significant social and healthcare stigma [[6], [7], [8], [9]]. This stigma manifests in both daily life and healthcare settings, where discrimination and harassment are prevalent [10]. Moreover, the reluctance to seek medical treatment due to fear of discrimination remains a significant barrier, contributing to higher rates of mental health issues, substance use, and certain health conditions, including sexually transmitted infections (STIs) [11]. While legislative changes have improved the visibility of LGBTQIA+ issues, the full integration of this community into mainstream healthcare systems remains a challenge. Consequently, the need for focused cancer care research within the LGBTQIA+ community is urgent to ensure that healthcare services are inclusive, equitable, and effective. This further aligns with the vision for health equity as outlined in the constitution of Nepal 2015, which aims to provide healthcare services to marginalized groups, including the LGBTQIA+ population. In this context, health equity ensures fair access to healthcare services based on individual needs, irrespective of sexual orientation or gender identity [12]. Healthcare disparities are characterized by differences in health outcomes and access to services, often influenced by systemic inequalities [13,14]. Nepal Cancer Control Strategy (NCCS) 2024–2030 also has set its objectives to reduce the incidence and prevalence of cancer through 9 different strategies which aims to promote the preventive, screening, curative, and palliative facilities thereby uplifting the quality of life of the cancer patients and affected family [15]. The strategy also aims to promote equitable distribution of oncology services through the country via manpower development and by empowering the existing private and governmental facilites [15].

This narrative review aims to address the knowledge gap by examining the specific barriers LGBTQIA+ individuals face in accessing equitable cancer care in Nepal. We explore how structural inequities, health care providers' biases, and gaps in cancer-specific education contribute to disparities throughout the cancer care continuum—from prevention to survivorship. The review concludes with strategic recommendations to advance inclusive and equitable oncology care for the LGBTQIA+ population in Nepal.

## 2 Methodology

A narrative review of existing literature was performed to summarize the evidence regarding cancer care related to LGBTQIA+ individuals from Nepal. Unlike a systematic review, which follows predefined inclusion criteria and assesses study quality quantitatively, a narrative review provides a broad summary of the available evidence, identifying patterns and gaps without conducting a formal meta-analysis [16,17]. Given the limited availability of Nepal-specific literature, this review also incorporates relevant studies from South-Asian countries and other LMICs with comparable socio-cultural and healthcare contexts to provide contextual understanding.

## 3 Search strategy

We conducted a comprehensive literature search using PubMed, Scopus, and Google Scholar databases. The search strategy included keywords such as “LGBTQIA+ health issues”, “sexual and gender minorities”, “risk factors”, “cancer care”, “Nepal healthcare,” “healthcare access”, “medical training,” and “stigma in healthcare”. The search was limited to articles published between 2010 and 2024 to ensure the inclusion of recent studies. Additionally, we reviewed reference lists of selected papers and relevant grey literature available from government, non-governmental, private, and academic/research institutions.

## 4 Inclusion/exclusion criteria

We included sources that focused on LGBTQIA+ populations and addressed one or more topics: cancer care, healthcare access, stigma and discrimination, provider training, meantal health or epidemiological trends, particularly within Nepal or similar LMIC settings. Studies were excluded if they lacked relevance to cancer care or LGBTQIA+ health, were purely theoretical without empirical basis, or focused on general population health without addressing sexual or gender minority status.

## 5 Analytical approach

This narrative review employed a structured thematic analysis following Braun and Clarke's six-step framework to organize and interpret the findings from existing literature on LGBTQIA+ health in the context of cancer care [18].

Our analytical process combined both deductive and inductive approaches. Initially, we applied a deductive coding framework based on pre-identified areas of interest such as healthcare access, stigma, risk factors, service delivery, and psychosocial support, grounded in the research questions and existing theoretical knowledge. Following this, we engaged in inductive coding to allow new themes and patterns to emerge directly from the data. This iterative process involved careful review and refinement of codes, facilitating the identification of themes that were both anticipated and novel within the context of LGBTQIA+ cancer care.

The final themes identified through this structured synthesis included: a lack of cancer-specific data and epidemiology; stigma and barriers to cancer screening and care; challenges within the health workforce, particularly the need for LGBTQIA+-inclusive training; elevated cancer risk factors among LGBTQIA+ populations; unmet mental health and psychosocial support needs; and significant gaps in policy, data systems, and community partnerships.

This structured synthesis provided a foundation to map challenges and propose targeted recommendations for improving equitable cancer care for LGBTQIA+ individuals in Nepal.

## 6 Results and discussion

**Fig. 1:**
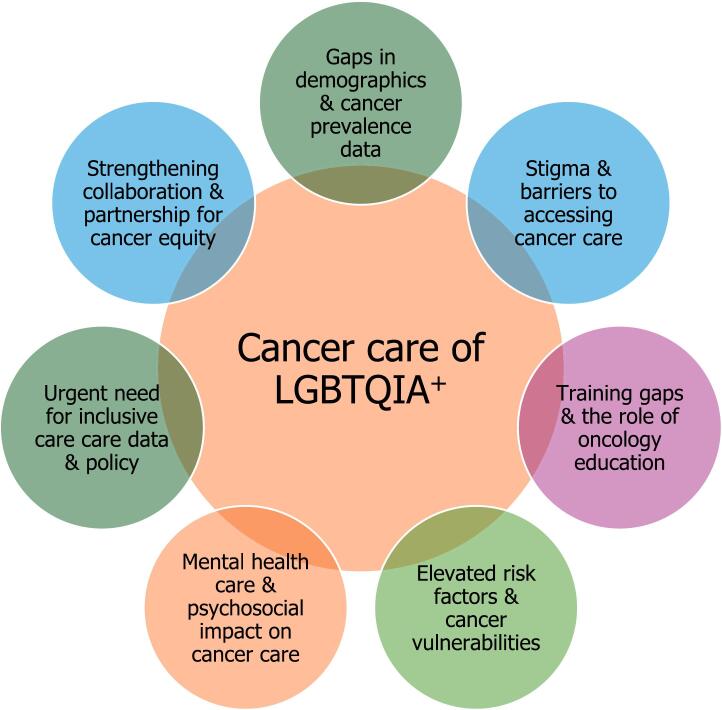
Themes focusing on Cancer care of LGBTQIA+ individual of Nepal.

This narrative review synthesizes literature across seven key themes (Fig. 1) that collectively shape the cancer care experience of LGBTQIA+ individuals in Nepal (1) gaps in demographic and cancer prevalence data, (2) stigma and barriers to accessing cancer care, (3) training gaps and the role of oncology education, (4) elevated risk factors and cancer vulnerabilities, (5) mental health care and psychosocial impact on cancer care (6) urgent need for inclusive care data and policy and (7) strengthening collaboration and partnership for cancer equity.

## 7 Gaps in demographics and cancer epidemiology data in the LGBTQIA+ population

According to Nepal's official statistics, ‘National Population and Housing Census 2021’, only 2928 out of approximately 30 million country's population were identified as LGBT individuals [19]. This figure is far below the two million population estimated by LGBTQIA+ rights groups in the country [20]. Despite Nepal's progressive legal framework, which emphasizes equitable rights for everyone, [21] social and cultural stigmatization continues to pose a significant barrier to identifying individuals as LGBTQIA+, thus leading to massive under-recording. Critically, Nepal's cancer registry does not collect data on sexual orientation or gender identity, making it impossible to assess the cancer burden or outcomes within this population [22]. This invisibility in data systems significantly impairs the development of evidence-based oncology policies and resource allocation for LGBTQIA+ individuals.

## 8 Stigma and barriers to accessing cancer care

Social stigma and discrimination remains a central barrier to cancer prevention and care in the LGBTQIA+ population [23]. This societal stigma manifests in various forms, such as family rejection, social ostracism, and verbal or physical abuse, including instances like verbal micro-aggressions during healthcare encounters (e.g., misgendering, insensitive comments, or dismissal of concerns), as well as physical abuse or exclusion from family support due to sexual orientation or gender identity [23,24]. These challenges lead to delays in seeking medical care, mistrust of healthcare providers, and a lower likelihood of utilizing preventive services such as cancer screening and human papillomavirus (HPV) vaccination [[25], [26], [27]]. Studies have reported lower screening rates and higher vaccination hesitancy among this population [28,29]. This marginalization potentially contributes to late-stage cancer diagnosis and poor prognosis [30]. In the context of neighboring countries such as India, transgender people have been reported to miss the cancer treatment process because of the existing taboo and lack of proper acceptance of the LGBTQIA+ population, [31] underscoring the need for further attention. A significant barrier to improving cancer outcomes among LGBTQIA^+^ individuals is limited access to cancer screening programs [32]. Discrimination and stigma not only deter individuals from seeking routine screenings but also contribute to later-stage cancer diagnoses, exacerbating health disparities [33,34]. For instance, fear of judgment or mistreatment by healthcare providers [35] often discourages LGBTQIA+ individuals from participating in screening programs. Expanding access to culturally competent and inclusive screening services is essential. Research shows that implementing targeted outreach, training healthcare providers on LGBTQIA+-specific needs, and creating supportive environments can increase screening uptake [36]. Moreover, integrating community-based initiatives and telehealth options can further improve access to preventive care.

## 9 Training gaps and the role of oncology education

Many healthcare professionals in Nepal receive little or no formal training on LGBTQIA+ health needs, particularly within oncology. A recent survey revealed that many LGBTQIA+ individuals felt uncomfortable discussing their sexual orientation or gender identity with their doctors due to fear of stigma [37]. This lack of open communication can result in inadequate reporting of medical histories, missed screenings, and suboptimal treatment plans. Literature shows poor knowledge and attitude of Nepalese medical students towards LGBTQIA+ community individuals [6,23,38]. Incorporating LGBTQIA+-inclusive oncology modules into medical and nursing curricula would ensure providers understand cancer-related risks, psychosocial challenges, and communication needs for diverse patients [6]. A comprehensive training program on equity and diversity is needed for healthcare providers in Nepal. This training needs to include modules covering unique health challenges faced by LGBTQIA+ individuals, such as specific cancer risk factors, mental health issues, and barriers to care. It should also focus on developing communication skills through training on empathetic and patient-centric communication, respecting privacy and confidentiality, and using inclusive language to foster a supportive environment. Additionally, practical exercises using real-life scenarios would help providers practice non-judgmental interactions and effective decision-making. Implementation strategies should also involve collaborating with local and international LGBTQIA+ organizations to create culturally relevant training materials. Integrating LGBTQIA+ health issues into the healthcare curriculum is crucial to ensure that future healthcare providers are well-prepared. Furthermore, offering workshops and online courses as part of ongoing professional development for current healthcare providers will provide continuous learning and improvement.

Establishing a feedback system where LGBTQIA+ patients can anonymously report their healthcare experiences will help refine and improve training programs over time. A recent report published by Astraea Lesbian Foundation for Justice highlighted perceived stigma and discrimination faced by the LGBTQIA+ community in healthcare settings, particularly from healthcare providers [39]. Providing targeted training on LGBTQIA+-specific health issues can improve the quality of care and reduce discrimination in healthcare settings [40]. This training should emphasize creating a welcoming and inclusive environment, normalizing discussions about mental health, and sharing experiences to build trust and understanding between patients and providers [41].

## 10 Elevated risk factors and cancer vulnerabilities

LGBTQIA+ individuals in Nepal experience higher rates of cancer-related behavioral and biological risk factors, including smoking, alcohol use, obesity, and HPV/STI exposure [[41], [42], [43], [44]]. Increased prevalence of certain risk factors among LGBTQIA+ in Nepal may heighten their vulnerability to specific types of cancer such as breast, cervical, anal, colorectal, lung, endometrial and prostate cancer [45,46]. Key risk factors include smoking, hazardous alcohol consumption, and exposure to HPV, which increase the likelihood of developing these cancers [44]. A study conducted among sexual and gender minorities in the Kathmandu Valley of Nepal showed a high prevalence of non-communicable disease risk factors, including insufficient fruits and vegetable consumption (95.7 %), current smoking (40 %), hazardous alcohol consumption (32.9 %), overweight/obesity (28.5 %), and hypertension (28.6 %) [47]. These are often linked to stress, social marginalization, and economic insecurity. Studies done elsewhere also report higher rates of tobacco and alcohol use, mental health issues, cardiovascular disease, and obesity among these populations [[48], [49], [50], [51], [52]]. Higher rates of smoking and alcohol consumption among LGBTQIA+ populations, for example, have a well-established risk of lung, liver, and oesophageal cancers. Risk factors are more prevalent, especially in those involved as sex workers [53]. Additionally, the increased prevalence of HPV and other STIs in the LGBTQIA^+^ community can significantly elevate the risk of cervical, anal, penile, and oropharyngeal cancers [33]. Existing literature on cancer prevalence and risk often lacks specific data on LGBTQIA+ individuals, highlighting a critical gap in understanding their unique healthcare needs [[54], [55], [56]]. Studies from global contexts indicate that men who have sex with men (MSM) and transgender individuals are at increased risk for HPV-related cancers due to factors such as unprotected sexual practices and limited access to preventive healthcare [57]. Despite these risks, access to HPV vaccination and cancer screening remains limited. Incorporating inclusive messaging in public health campaigns, ensuring availability of gender-neutral vaccination strategies, and creating safe spaces for routine cancer screenings are essential interventions.

## 11 Mental health care and psychosocial impact on cancer care

Access to mental health care is limited for the general population in Nepal, [58] and this issue is even more profound for LGBTQIA+ individuals. Mental health issues are reported to be more prevalent within the LGBTQIA+ community due to the stress of coping with societal discrimination and rejection [59]. In Nepal, MSM are particularly affected, often experiencing exclusion from mental health services [60]. A study from Nepal revealed high prevalence rates of stress (82.3 %), anxiety (65.2 %), and depression (74.1 %) among sexual and gender minorities. Additionally, 88.6 % of participants reported experiencing discrimination during their lifetime [61]. A cross-sectional study investigating suicidal ideation, plans and attempts among MSM found a prevalence of 42.4 % for suicidal ideation, 31.2 % for plans and 21.6 % for attempts [62]. Literature underscores the need for earlier detection of suicidal behaviour, and the development of interventions such as mobile apps to improve access to mental health care services [60,62]. These mental health challenges, including depression, anxiety, and substance abuse, can indirectly impact cancer outcomes by affecting individuals' ability to seek timely medical care and adhere to treatment regimens [63,64]. Stress, anxiety, depression, and suicide ideation found to beprevalent in LGBTQIA+ communities due to ongoing discrimination can interfere with treatment adherence, decision-making, and health-seeking behaviors [[58], [59], [60], [61], [62], [63]].

For patients with cancer, unmanaged mental health concerns can negatively influence the quality of life and survival outcomes [[64], [65], [66], [67]]. Integrating psychosocial support within oncology services is a cost-effective and necessary strategy. This may include training oncology teams in LGBTQIA+-affirming care, creating peer support groups, and expanding telehealth counseling services to reach rural and marginalized communities.

## 12 The urgent need for inclusive cancer data and policy

A core challenge in addressing cancer disparities in the LGBTQIA+ population is the absence of inclusive cancer surveillance data. Nepal currently lacks mechanisms to collect sexual orientation and gender identity (SOGI) data in cancer registries, health surveys, or hospital systems [6,9]. Without such data, inequities remain hidden and unaddressed. It is, therefore, crucial to increase the reporting of sexual orientation and gender identity in national cancer registries and health surveys. This will provide evidence-based insights to drive targeted interventions and resource allocation for cancer prevention and treatment programs. The importance of collecting such data cannot be overstated. Tracking sexual orientation and gender identity in national health surveys and cancer registries allows for the identification of disparities, monitoring progress over time, and ensuring that resources are directed to where they are most needed [65,66]. However, collecting this data presents several challenges, including privacy concerns and fear of discrimination. It is essential to create a stigma-free environment and ensure confidentiality to encourage individuals to disclose their sexual orientation and gender identity. Furthermore, training data collectors and healthcare providers on the significance of collecting this information respectfully and sensitively is vital to improving data quality and participation rates.

This lack of data on LGBTQIA+ individuals underscores the need for policy changes that mandate the inclusion of sexual orientation and gender identity in health surveys and cancer registries. Such policy reforms would ensure that data collection is standardized and inclusive, ultimately improving health outcomes for LGBTQIA+ individuals.

## 13 Strengthening collaboration and partnerships for cancer equity

Civil society organizations in Nepal play a vital role in supporting LGBTQIA+ health. While many LGBTQIA+ individuals rely on voluntary and community organizations for healthcare support, reports suggest that strengthening and aligning with these organizations through funding, capacity-building, and partnerships can help minimize the risks and increase health-related awareness [[67], [68], [69], [70]]. In Nepal, several organizations, including Blue Diamond Society [71], Federation of Sexual and Gender Minorities-Nepal [72], *Mitini* Nepal [73], *Sparsha* Nepal [74], and Cruise Aids Nepal play a crucial role in advocating for LGBTQIA+ health rights and providing essential services. Strengthening collaborations between these organizations, government agencies, and international agencies can enhance cancer research, improve healthcare access, and promote culturally competent care. Such partnerships can facilitate resource-sharing, support policy implementation, and ensure a more inclusive approach to cancer prevention and treatment for LGBTQIA+ individuals in Nepal.

## 14 Limitations of this study

This narrative review is constrained by the lack of disaggregated data specific to LGBTQIA+ individuals within Nepal's routine healthcare systems. The review relied heavily on studies from neighboring countries and international literature, which may limit contextual accuracy. Additionally, as a narrative review, the study synthesis was not conducted through systematic review protocols, potentially introducing selection bias.

## 15 Conclusion

LGBTQIA+ individuals in Nepal face distinct and underrecognized challenges across the cancer care continuum, including a lack of disaggregated data on cancer prevalence, limited access to screening and treatment, elevated exposure to behavioral and biological risk factors, and widespread stigma within healthcare settings. These disparities are compounded by insufficient training among healthcare providers and a lack of culturally competent oncology services. Partnerships with LGBTQIA+ organizations, LGBTQIA+ community outreach awareness programs, and inclusive policy reforms are vital to ensuring that Nepal's cancer care system aligns with the constitutional commitment to equity and non-discrimination. Efforts should prioritize inclusive, evidence-based interventions that address the unique cancer risk factors and specific care needs of the LGBTQIA+ population in Nepal. Prioritizing this concern further aligns with the objectives set out by the NCCS 2024–2030.

## Author contribution

S·S. conceptualized and led the overall project, contributed to writing the initial draft, and critically reviewed and revised the manuscript for intellectual content. N.P. contributed to the manuscript's design and conceptual framework, conducted literature review, added contents, provided critical revisions, and shaped the discussion on healthcare equity. S.Sa, ST, SP, PB, and JB collaboratively drafted sections on cancer research gaps, provided input on research priorities, contributed to the literature review and analysis, and refined the manuscript, with a focus on LGBTQIA+ healthcare gaps, data interpretation, and addressing LGBTQIA+ community needs in Nepal. P.K. and K.R. contributed to public health policy, manuscript design, and draft clarity while shaping the conceptual framework, providing healthcare insights, and ensuring consistency. V.P. and D.S. contributed to the literature review, manuscript writing, and editing, and offered critical insights to identify practical solutions for equitable cancer care, while helping finalize the manuscript. All authors agreed on the final version of the manuscript.

## CRediT authorship contribution statement

**Sunil Shrestha:** Writing – review & editing, Writing – original draft, Visualization, Validation, Supervision, Methodology, Conceptualization. **Nabin Pathak:** Writing – review & editing, Writing – original draft, Visualization, Conceptualization. **Simit Sapkota:** Writing – review & editing, Resources, Project administration, Investigation, Conceptualization. **Sudip Thapa:** Writing – review & editing, Resources, Conceptualization. **Subhas Pandit:** Writing – review & editing, Conceptualization. **Jeebana Bhandari:** Writing – review & editing, Methodology, Investigation, Funding acquisition, Formal analysis. **Pankaj Barman:** Writing – review & editing, Visualization, Validation. **Pratik Khanal:** Writing – review & editing, Visualization, Resources, Project administration, Conceptualization. **Kamal Ranabhat:** Writing – review & editing, Visualization, Validation. **Vibhu Paudyal:** Writing – review & editing, Validation, Supervision, Software, Resources. **Deependra Singh:** Writing – review & editing, Visualization, Supervision.

## Disclaimer

Where authors are identified as personnel of the International Agency for Research on Cancer/WHO, the authors alone are responsible for the views expressed in this article, and they do not necessarily represent the decisions, policy, or views of the International Agency for Research on Cancer/WHO.

## Funding

None.

## Declaration of competing interest

The authors declare that they have no known competing financial interests or personal relationships that could have appeared to influence the work reported in this paper.
